# Maternal Factors Associated with the Initiation of Exclusive Breastfeeding among Mothers at One Week after Delivery in Two Selected Hospitals in Kelantan, Malaysia

**DOI:** 10.21315/mjms2018.25.4.11

**Published:** 2018-08-30

**Authors:** Che Muzaini Che’ Muda, Tengku Alina Tengku Ismail, Rohana Ab Jalil, Suhaily Mohd Hairon, Zaharah Sulaiman, Nazirah Johar

**Affiliations:** 1 Department of Community Medicine, School of Medical Sciences, Universiti Sains Malaysia, 16150 Kubang Kerian, Kelantan, Malaysia; 2 Kedah State Health Office, Simpang Kuala, Jalan Kuala Kedah, 05400 Alor Setar, Kedah, Malaysia; 3 Women’s Health Development Unit, School of Medical Sciences, Universiti Sains Malaysia, 16150 Kubang Kerian, Kelantan, Malaysia; 4 Lactation Secretariat, Hospital Universiti Sains Malaysia, 16150 Kubang Kerian, Kelantan, Malaysia

**Keywords:** exclusive breastfeeding, post-partum, knowledge, attitude

## Abstract

**Background:**

The first week following delivery usually coincides with the initiation of exclusive breastfeeding. This study aimed to determine the prevalence of and the associated factors regarding the initiation of exclusive breastfeeding among mothers at one week after delivery in two selected hospitals in the state of Kelantan, Malaysia.

**Methods:**

A cross-sectional study was conducted from March to August 2015 among post-partum mothers, who were selected through systematic sampling. A newly developed and validated questionnaire on the participants’ data, knowledge and attitude items and a breastfeeding practice checklist were used. The mothers were interviewed in the post-natal ward, and their breastfeeding practices were determined through a phone call at one week following delivery. Descriptive statistics and simple and multiple logistic regressions were used for the data analysis.

**Results:**

A total of 335 participants were included. The prevalence of exclusive breastfeeding at one week post-partum was 77.9% (95% CI: 73.0%, 82.2%), with significant associated factors being previous exclusive breastfeeding experience [adjusted odds ratio (AOR) 2.48; 95% CI: 1.37, 4.49; *P*-value = 0.003] and the mean total score of knowledge [AOR 1.06; 95% CI: 1.01, 1.11; *P*-value = 0.011].

**Conclusion:**

Every mother should receive breastfeeding education, with special emphasis on those with no previous experience. The weak areas of knowledge identified herein should be strengthened during health education.

## Introduction

The standard recommendation is that infants should be exclusively breastfed for the first six months of life, with the introduction of complementary food at the age of six months, and that breastfeeding should continue for at least two years (1, 2). Breastfeeding provides many benefits to the infant, mother and family, including the infant’s protection against diarrhoeal diseases, respiratory infection, overweight and obesity and the mother’s protection against post-partum haemorrhage and short birth spacing (3–7).

Despite the well-recognised importance of exclusive breastfeeding, the practice is not widespread, neither globally nor in Malaysia. By increasing the coverage of exclusive breastfeeding up to 90.0%, it has been estimated that 1.30 to 1.45 million child deaths in 42 high-mortality countries could be prevented (8). Although exclusive breastfeeding rates among infants aged six months attending government health clinics in Malaysia increased to 23.3% in 2011 from 14.5% in 2006, the rate is still far below the standard recommendation (9, 10).

Breastfeeding involves the initiation and maintenance of behaviour. The first week following delivery is a critical period, as postpartum mothers are in the midst of initiating breastfeeding (11). This is the time when lactation is being established, and the mother and infant are learning how to feed the infant at the breast. Physiologically, the transitional milk, which is higher in quantity, is produced about four to six days after delivery. The infant’s feeding requirements also increase during this period (12). Frequent difficulties include breast engorgement, leaking of milk, fatigue from childbirth, perceived insufficient milk supply, and as a crying or sleepy infant may hinder the maintenance of lactation, formula feeding may be seen as an attractive option (13).

It is important to understand the factors that influence exclusive breastfeeding initiation and maintenance. Exclusive breastfeeding practice in the first week following delivery reflects the initiation of breastfeeding (11). Age, marital status, education and income level are the major socio-demographic factors affecting exclusive breastfeeding behaviour, and they are not easily influenced or modified (14, 15). Knowledge and attitudes are important modifiable factors that may improve exclusive breastfeeding practice (16, 17). This study aimed to determine the prevalence of and associated factors regarding the initiation of exclusive breastfeeding among mothers at one week following delivery in two selected hospitals in the state of Kelantan, Malaysia.

## Materials and Methods

### Study Design and Participants

A cross-sectional study was conducted from 1 March 2015 to 31 August 2015 among postpartum mothers who delivered a healthy infant and were able to initiate breastfeeding while in the post-natal wards of two district hospitals in the state of Kelantan, Malaysia. Hospital Pasir Mas and Hospital Tengku Anis were purposively chosen for this study, as they were both recipients of the Baby Friendly Hospital Award under Baby Friendly Hospital Initiative (BFHI), and they offered normal delivery services for low-risk pregnant mothers. Those who were medically contraindicated to breastfeeding were excluded from the study.

Sample size was calculated using power and sample size (PS) software for single and two proportions, with a type I error of 5% and a type II error of 20%. The largest calculated sample size was 303; when calculated for a single-proportion formula and after a 10% non-response consideration, the required sample size was 336. Ethical approval was obtained from Universiti Sains Malaysia and the Ministry of Health.

### Data Collection

A systematic sampling was conducted on every data collection day. After considering the interview duration and the length of hospital stay prior to discharge, every alternate mother in the ward admission list was selected so as to reach the total of three participants per day. Each participant’s written informed consent was obtained, and details of addresses and contact numbers were recorded. The newly developed and validated Knowledge, Attitude and Practice Breastfeeding (KAP-BF) questionnaire was used in this study. The participants answered two parts of the interviewer-guided questionnaire while in the hospital, which were i) participants’ data and ii) knowledge and attitude items. Then, at one week following delivery, their breastfeeding practices were determined through a phone call and recorded in the breastfeeding practice checklist.

### Knowledge, Attitude and Practice Breastfeeding (KAP-BF) Questionnaire

The KAP-BF questionnaire consisted of three parts: i) participants’ data, ii) knowledge and attitude items and iii) the breastfeeding practice checklist. A team comprised of a public health physician, a breastfeeding consultant, a breastfeeding counsellor, a nutritionist and a biostatistician were involved in the development of the KAP-BF questionnaire.

The participants’ data consisted of socio-demographic characteristics, medical history, delivery history and breastfeeding history. The knowledge questionnaire was adapted from a Malay version of the Breastfeeding Knowledge questionnaire, which was validated among female university staff (18). It contained 41 items covering breastfeeding knowledge such as the advantages of breastfeeding to the baby and mother, colostrum, effective feeding, breast milk expression, duration of feeding, complementary feeding, problems with breastfeeding, breast engorgement and practical aspects of breastfeeding. A correct answer scored as “1”, while an incorrect or unsure answer scored as “0”. Items with reverse statements were scored accordingly.

The items on attitude were developed in stages. The first stage involved searching the literature that most related to our study population, followed by series of discussions with the team members to discuss the items and to verify their content validity. The items were distributed over three sub-domains covering cognitive, affective and behavioural aspects of attitude. The attitude score was recorded from 1 to 5 for “strongly agree”, “agree”, “unsure”, “disagree” and “strongly disagree”, respectively, while for items with negatively arranged responses, the scores were reversed. Therefore, all items with higher scores reflected a more positive response to attitude towards breastfeeding. The scores for each item of knowledge and attitude domain were summed to create an overall score for each domain.

A validation study was conducted among post-partum mothers in a teaching hospital in Kelantan. Item analyses of the knowledge domain had an acceptable difficulty index, with an excellent discrimination index. As exploratory factor analysis constructed for attitude domain loaded from 0.53 to 0.86, with a total of 11 items. Cronbach’s alpha of the final questionnaire was 0.85 for knowledge and 0.79 for attitude.

The breastfeeding practice checklist included self-reported practices of breastfeeding or feeding with expressed breast milk, water feeding, formula feeding and complementary feeding. An option of “never”, “seldom” or “always” was applied for each practice. The option “never” was defined as never feeding the infant with the specific feeding method; “seldom” was defined as feeding the infant with the specific feeding method for less than seven days per week; and “always” was defined as feeding the infant with the specific feeding method every day. In this study, exclusive breastfeeding refers to feeding an infant with breast milk from his or her mother, and/or expressed breast milk, without any additional solid or liquid, except for oral rehydration salt, drops, syrups of vitamins and minerals or medicines (19).

### Statistical Analysis

Data entry and statistical analyses were performed using the SPSS software version 22.0 (20). The analysis was started with a data exploration to check for data normality and potential errors in data entry. The data were presented as a mean and standard deviation (SD) for the numerical variables, with frequency (percentages) for the categorical variables.

Statistical analyses were conducted using simple and multiple logistic regressions. The outcome variable was exclusive breastfeeding at one week after delivery. Simple logistic regressions were used to select the preliminary variables regarding association with exclusive breastfeeding at one week after delivery. Variables with a *P-*value of less than 0.25 or any clinically relevant and important variable were included in the multiple logistic regression analysis. Multiple logistic regressions were used to evaluate factors associated with exclusive breastfeeding at one week after delivery. A preliminary main effect model was obtained after comparing the model using Backward Likelihood Ratio (LR) and Forward LR methods. The fitness of the model was tested using the Hosmer and Lemershow goodness of fit test, the classification table and the receiver operating characteristic (ROC) curve. The significance level was set at 0.05.

## Results

A total of 335 participants were included, with a response rate of 86% at one week after delivery. All the participants were Malay, Muslim and married. Their socio-demographic characteristics are presented in Table 1. The mean (SD) age was 28.64 (5.30) years. Half of them were housewives and multiparous. Table 2 shows the participants’ delivery history. The majority of them had practiced skin-to-skin contact with their infants after delivery and were able to initiate breastfeeding within the first hour. However, only 184 participants (54.9%) had a companion during delivery.

Table 3 shows the participants’ breastfeeding history. Less than half of the participants, 151 (45.1%) had previous experience of exclusive breastfeeding for six months. Altogether, 311 participants (92.8%) had an intention to exclusively breastfeed their newborn infants until six months, and 70% of them received antenatal health education on breastfeeding.

Of the 41 items in the knowledge domain and 11 items in the attitude domain, the maximum score was 41 and 55 for knowledge and attitude, respectively. Both the knowledge and attitude scores were normally distributed, and the mean (SD) total scores of knowledge and attitude among the participants were 29.63 (6.07) and 44.12 (4.13), respectively. The lowest correct responses, which were about 40.0%, concerned breast engorgement. The correct responses on the items relating to knowledge on breastmilk expression ranged from 40.0% to 74.0%.

The prevalence of exclusive breastfeeding at one week after delivery was 77.9% (95% CI: 73.0%, 82.2%). Table 4 shows the participants’ breastfeeding practice at one week after delivery. Among 74 participants who did not practice exclusive breastfeeding, two of them exclusively formula-fed their infants.

A simple logistic regression analysis, which was used to determine exclusive breastfeeding practice, identified seven variables with *P* < 0.25. These variables were included in the multiple logistic regression analysis, with the addition of one clinically significant variable, and two variables were found to be significantly associated with exclusive breastfeeding at one week after delivery at the multivariable level (Table 5).

The logistic regression model revealed the following: participants with previous exclusive breastfeeding experience had 2.48 times the odds of exclusively breastfeeding their infants at one week after delivery compared to those who had no experience of exclusive breastfeeding (95% CI: 1.37, 4.49; *P*-value = 0.003). Participants with an increase of one unit of the mean total score of knowledge had 1.06 times the odds of exclusively breastfeeding their infants at one week after delivery (95% CI: 1.01, 1.11; *P*-value = 0.011).

## Discussion

The prevalence of exclusive breastfeeding at one week after delivery was 77.9%, which was slightly lower than a study in rural Vietnam, with 83.6% of exclusive breastfeeding at one week (13). A population-based longitudinal birth cohort study in south-western Sweden found that 74.0% of infants were exclusively breastfed at one week after delivery (11). In southwestern Ontario, 68.0% of infants were fully breastfed at one week after delivery (21), and in San Francisco, California, 79.1% were exclusively breastfed at four days after delivery (22).

At one week after delivery, 15.2% of the participants also fed their infants water, 11.3% gave formula feeding to their infants, and two participants exclusively formula-fed their infants. In their study, Duong et al. (13) reported about 7.0% of mothers introduced formula to their infants at one week, including eight mothers who exclusively bottle fed. The reasons included suggestions from relatives or friends, the ease of bottle feeding, lack of enjoyment regarding breastfeeding and working schedules.

A study in Brazil found that almost 6% of infants were given water on the first day after hospital discharge (23). Almost one-third of mothers in Ghana gave water to their infants after delivery (24). Even in the obstetric ward, 1.7% of mothers offered water to their infants, 6.9% formula milk and 1.1% offered tea or coffee (25). Breast milk contains 88.0% water and is low in solute (26). Adding water in the early days after delivery is associated with the increased risk of early cessation of breastfeeding (27). The choice of combination feeding might indicate lower certainty among mothers, whereby mothers who stopped breastfeeding within one week reported significantly less certainty in the decision to breastfeed and a more positive attitude towards human-milk-substitute feeding (28).

Participants with previous exclusive breastfeeding experience had 2.48 times the odds of exclusively breastfeeding their infants at one week after delivery compared to those who had no experience of exclusive breastfeeding. Previous exclusive breastfeeding experience might assist mothers in building confidence to exclusively breastfeed. Exclusive breastfeeding for six months is indeed challenging. Those who were able to practice this before were more capable of overcoming breastfeeding difficulties in the first week following delivery. Mothers with little or no previous breastfeeding experience required additional support to be able to breastfeed adequately (29). However, a study by Almqvist-Tangen et al. (11) found no association of previous exclusive breastfeeding experience with exclusive breastfeeding at one week after delivery.

In contrast, a study by Clifford et al. (21) found that mothers who did not have previous breastfeeding experience, either because they were primiparas or because they were multiparas who had not breastfed a previous child/children, were significantly more likely to breastfeed exclusively at one week than those who had previous breastfeeding experience. It might also be that the demands of caring for a larger family are perceived by multiparas with previous breastfeeding experience to place them at a disadvantage if they choose to breastfeed the current infant.

Participants with an increase of one unit of the mean total score of knowledge had 1.06 times the odds of exclusively breastfeeding their infants at one week after delivery. Mothers with adequate knowledge on exclusive breastfeeding were more capable of overcoming breastfeeding difficulties, especially in the early period after delivery (17). Those who understood the physiological changes in milk production were less likely to have any perceived insufficient milk supply, which was among the common reasons given for discontinuing exclusive breastfeeding. Breastfeeding problems in the first few days following delivery concerned factors associated with the discontinuation of breastfeeding at one week after delivery (11); however, if mothers had the knowledge of ways to overcome these problems, most of them could breastfeed as recommended.

Our study noted a lack of knowledge regarding breast milk expression. Ekambaram et al. (30) also noted a similar finding; in their study, 51.0% answered correctly regarding expressed breastmilk, 34.0% on the techniques of expressing breastmilk and 2.0% on the storage of expressed breastmilk at room temperature. Mothers need to know how to express their milk so that they can continue to feed their infants and keep up their milk supply if they are separated from their infants. This is a fundamental element, taking into account the increasing proportion of working mothers who need to be taught during the ante-natal or postnatal periods by healthcare workers. Similarly, a lack of knowledge in managing breast engorgement will lead to early breastfeeding cessation, as it influences milk supply. Breast engorgement may be due to latching difficulties or clogged milk ducts (31).

In our study, the majority of participants had the intention to exclusively breastfeed their infants until six months. Intention to breastfeed is one of the most important factors in enhancing and prolonging breastfeeding. A community-based epidemiological study on breastfeeding and associated factors with respect to post-partum periods in Taiwan found that mothers who had the intention to breastfeed their infants before delivery had the odds ratios of breastfeeding increased in the postpartum periods from 3.0 (in hospital stay), 6.1 (at one month post-partum) and 13.9 (at four months post-partum) to 16.8 (at six months post-partum) (32). Although there was a strong positive association between planned and actual breastfeeding duration, this relationship was modifiable. Regardless of the woman’s initial intentions, promotional and educational strategies were effective at increasing her duration of lactation (33).

Support during delivery is important, but only half of the participants had a companion during delivery. It is the policy of the Ministry of Health to promote a husband-friendly environment when the mother is in the active phase of delivery. In University College Hospital, Ibadan, Nigeria, 55.0% of first-time mothers had a companion during delivery, and the median time to breastfeeding initiation was significantly shorter among those with companions compared to those without (*P* < 0.01) (34). This finding might be explained by psychological encouragement and assistance by companions while women were still experiencing post-delivery pain. The majority of our participants performed skin-to-skin contact after delivery and were able to breastfeed their infants within one hour of delivery. Both hospitals received the Baby Friendly Hospital Award under the Baby Friendly Hospital Initiative (BFHI), which has contributed to the practices. Moreover, time to breastfeeding initiation was one of the most commonly reported independent predictors of exclusive breastfeeding in many communities (35).

## Conclusion

The prevalence of exclusive breastfeeding at one week after delivery among mothers who delivered at two district hospitals in the state of Kelantan, Malaysia, was not satisfactory. Strategies or intervention should be aimed at the identified factors associated with exclusive breastfeeding at one week, previous exclusive breastfeeding experience and mean total scores of knowledge. Every woman should receive education and support to practice exclusive breastfeeding, especially targeting those with no prior experience, who have an increased risk of early weaning. Targeting programmes should be given more attention and focus so as to increase knowledge among mothers, especially in the area of problems regarding breastfeeding, such as breast engorgement and breast milk expression, because this will help mothers successfully manage the breastfeeding problems they encounter in the early period following delivery, thus sustaining the practice of exclusive breastfeeding.

## Figures and Tables

**Table 1:** Socio-demographic characteristics and medical history of the participants (*n* = 335)

Variables	Frequency (%)	Mean (SD)
Age (year)		28.64 (5.30)
Educational level		
None/Primary school	8 (2.4)	
Secondary school	214 (63.9)	
Tertiary education	113 (33.7)	
Occupation		
Housewife	194 (57.9)	
Self-employed	26 (7.8)	
Government sector	78 (23.3)	
Private sector	37 (11.0)	
Husband’s educational level		
None/Primary school	15 (4.5)	
Secondary school	233 (69.5)	
Tertiary education	87 (26.0)	
Husband’s occupation		
Unemployed	5 (1.5)	
Self-employed	164 (49.0)	
Government sector	84 (25.0)	
Private sector	82 (24.5)	
Household income (RM)		2469.48 (1950.47)
Parity		
Primiparous	108 (32.3)	
Multiparous	190 (56.7)	
Grandmultiparous	37 (11.0)	
Co-morbidities*		
Pregnancy induced hypertension	5 (1.5)	
Gestational diabetes mellitus	18 (5.4)	

* non-mutually exclusive

**Table 2:** Delivery history of the participants (*n* = 335)

Variables	Frequency (%)
Infant’s sex	
Male	158 (47.2)
Female	177 (52.8)
Companion during delivery	
No	151 (45.1)
Yes	184 (54.9)
Person accompanied (*n* = 184)	
Husband	149 (44.5)
Mother	29 (8.6)
Mother-in-law	2 (0.6)
Sister	2 (0.6)
Sister-in-law	2 (0.6)
Skin-to-skin contact	
No	22 (6.6)
Yes	313 (93.4)
Breastfeeding within one hour after delivery	
No	14 (4.2)
Yes	321 (95.8)

**Table 3:** Breastfeeding history of the participants (*n* = 335)

Variables	Frequency (%)
Exclusive breastfeeding experience	
No	184 (54.9)
Yes	151 (45.1)
Intention of exclusive breastfeeding up to six months	
No	24 (7.2)
Yes	311 (92.8)
Ever joining any breastfeeding support group during pregnancy	
No	241 (71.9)
Yes	94 (28.1)
Ever joining any breastfeeding classes during pregnancy	
No	298 (89.0)
Yes	37 (11.0)
Ever receiving health education on breastfeeding during pregnancy	
No	77 (23.0)
Yes	258 (77.0)

* Statement 2 to 5 refers to recent pregnancy

**Table 4:** Participants’ breastfeeding practice at one week after delivery (*n* = 335)

Variables	Frequency (%)	95% CI
Exclusive breastfeeding	261 (77.9)	73.0, 82.2
Non-exclusive breastfeeding	74 (22.1)	17.8, 26.9
Feeding methods other than exclusive breastfeeding (*n* = 74)		
Breastfeeding with water feeding	34 (10.1)	
Breastfeeding with formula milk feeding	24 (7.2)	
Breastfeeding with water and formula milk feeding	14 (4.2)	
Formula milk feeding only	2 (0.6)	

**Table 5:** Factors associated with exclusive breastfeeding at one week after delivery (*n* = 335)

Variables	Crude OR^a^ (95% CI)	Adjusted OR^b^ (95% CI)	Wald Stat^a^ (df)	*P*-value^a^
Age (year)	1.04 (0.99, 1.10)		2.65 (1)	0.103
Husband’s age (year)	1.03 (0.99, 1.07)		1.97 (1)	0.161
Parity				
Primiparous	1			
Multiparous	2.89 (1.65, 5.07)		13.80 (2)	< 0.001
Grandmultiparous	1.89 (0.79, 4.54)		2.01 (2)	0.156
Companion during delivery				
No	1			
Yes	1.59 (0.95, 2.67)		3.07 (1)	0.080
Skin to skin contact				
No	1			
Yes	0.16 (0.02, 1.18)		3.22 (1)	0.072
Previous exclusive breastfeeding experience				
No	1	1		
Yes	2.96 (1.67, 5.27)	2.48 (1.37, 4,49)	8.97 (1) b	0.003^b^
Mean total score of knowledge	1.08 (1.03, 1.13)	1.06 (1.01, 1.11)	6.40 (1) b	0.011^b^
Mean total score of attitude	1.03 (0.97, 1.10)		1.04 (1)	0.307

a Simple logistic regression

b Multiple logistic regression

Backward LR method used

The Hosmer-Lemeshow goodness-of-fit test,*P*-value = 0.836

Classification table = 78.2%

The area under ROC curve = 0.67, *P*-value < 0.001

There are no interaction and multicollinearity problems

## References

[1] National Coordinating Committee on Food and Nutrition (2010). Malaysian dietary guidelines.

[2] World Health Organization (2003). Global strategy for infant and young child feeding.

[3] Al Mamun A, O’Callaghan MJ, Williams GM, Najman JM, Callaway L, McIntyre HD (2015). Breastfeeding is protective to diabetes risk in young adults: a longitudinal study. Acta Diabetol.

[4] Anatolitou F (2012). Human milk benefits and breastfeeding. Journal of Pediatric and Neonatal Individualized Medicine.

[5] Ip S, Chung M, Raman G, Chew P, Magula N, De Vine D (2007). Breastfeeding and maternal and infant health outcomes in developed countries. Evid Report Technol Assess (Full Rep).

[6] Labbok MH (2015). Postpartum sexuality and the lactational amenorrhea method for contraception. Clinical Obstetrics and Gynecology.

[7] Zaharah S, Lisa HA, Liamputtong P (2012). Infant feeding practices: rates, risks of not breastfeeding, and factors influencing. Breastfeeding evolution, early experience and human development: from research to practice and policy.

[8] Jones G, Steketee RW, Black RE, Bhutta ZA, Morris SS, Group BCSS (2003). How many child deaths can we prevent this year?. Lancet.

[9] Fatimah SJ, Siti Saadiah HN, Tahir A, Hussain Imam MI, Ahmad Faudzi Y (2010). Breastfeeding in Malaysia: results of the Third National Health and Morbidity Survey (NHMS III) 2006. Malays J Nutr.

[10] Ministry of Health Malaysia (2011). Annual report 2011.

[11] Almqvist-Tangen G, Bergman S, Dahlgren J, Roswall J, Alm B (2012). Factors associated with discontinuation of breastfeeding before 1 month of age. Acta Paediatr.

[12] Pang WW, Hartmann PE (2007). Initiation of human lactation: secretory differentiation and secretory activation. J Mammary Gland Biol Neoplasia.

[13] Duong DV, Binns CW, Lee AH (2004). Breast-feeding initiation and exclusive breast-feeding in rural Vietnam. Public Health Nutr.

[14] Brand E, Kothari C, Stark MA (2011). Factors related to breastfeeding discontinuation between hospital discharge and 2 weeks postpartum. J Perinat Edu.

[15] Jones JR, Kogan MD, Singh GK, Dee DL, Grummer-Strawn LM (2011). Factors associated with exclusive breastfeeding in the United States. Pediatrics.

[16] De Jager E, Skouteris H, Broadbent J, Amir L, Mellor K (2013). Psychosocial correlates of exclusive breastfeeding: a systematic review. Midwifery.

[17] Tan K (2009). Knowledge, attitude and practice on breastfeeding in Klang, Malaysia. International Medical Journal.

[18] Tengku Alina TI, Zaharah S (2010). Reliability and validity of a Malay-version questionnaire assessing knowledge of breastfeeding. Malays J Med Sci.

[19] World Health Organization (2008). Indicators for assessing infant and young child feeding practices. Part I: definitions.

[20] IBM Corp (2013). IBM SPSS statistics for Windows, version 220.

[21] Clifford TJ, Campbell MK, Speechley KN, Gorodzinsky F (2006). Factors influencing full breastfeeding in a southwestern Ontario community: assessments at 1 week and at 6 months postpartum. J Hum Lact.

[22] Wojcicki JM, Gugig R, Tran C, Kathiravan S, Holbrook K, Heyman MB (2010). Early exclusive breastfeeding and maternal attitudes towards infant feeding in a population of new mothers in San Francisco, California. Breastfeed Med.

[23] Venancio SI, Saldiva SRDM, Mondini L, Levy RB, Escuder MML (2008). Early interruption of exclusive breastfeeding and associated factors, State of Sao Paulo, Brazil. J Hum Lact.

[24] Singh B (2010). Knowledge, attitude and practice of breastfeeding–a case study. European Journal of Scientific Research.

[25] Shwetal B, Pooja P, Neha K, Amit D, Rahul P (2012). Knowledge, attitude and practice of postnatal mothers for early initiation of breast feeding in the obstetric wards of a tertiary care hospital of Vadodara city. National Journal of Community Medicine.

[26] Lawrence RA, Lawrence RM (2005). Breastfeeding: A guide for the medical profession.

[27] Becker GE, Remmington S, Remmington T (2011). Early additional food and fl uids for healthy breastfed full-term infants (review).

[28] Chezem JC, Friesen C, Boettcher J (2003). Breastfeeding knowledge, breastfeeding confidence, and infant feeding plans: effects on actual feeding practices. JOGN Nurs.

[29] Kronborg H, Væth M, Olsen J, Iversen L, Harder I (2007). Effect of early postnatal breastfeeding support: a cluster-randomized community based trial. Acta Paediatr.

[30] Ekambaram M, Bhat B, Vishnu B, Padiyath AM (2010). Knowledge, attitude and practice of breastfeeding among postnatal mothers. Current Pediatric Research.

[31] Avery A, Zimmerman K, Underwood PW, Magnus JH (2009). Confident commitment is a key factor for sustained breastfeeding. Birth.

[32] Kuo SC, Hsu CH, Li CY, Lin KC, Chen CH, Gau ML (2008). Community-based epidemiological study on breastfeeding and associated factors with respect to postpartum periods in Taiwan. J Clin Nurs.

[33] Kramer MS, Chalmers B, Hodnett ED, Sevkovskaya Z, Dzikovich I, Shapiro S (2001). Promotion of breastfeeding intervention trial (PROBIT). A randomized trial in the Republic of Belarus. The Journal of the American Medical Association.

[34] Morhason-Bello IO, Adedokun BO, Ojengbede OA (2009). Social support during childbirth as a catalyst for early breastfeeding initiation for first-time Nigerian mothers. Int Breastfeed J.

[35] Forster DA, McLachlan HL (2007). Breastfeeding initiation and birth setting practices: a review of the literature. J Midwifery & Women's Health.

